# A novel endothelial-related prognostic index by integrating single-cell and bulk RNA sequencing data for patients with kidney renal clear cell carcinoma

**DOI:** 10.3389/fgene.2023.1096491

**Published:** 2023-03-10

**Authors:** Deng-Xiong Li, Qing-Xin Yu, Chui-Xuan Zeng, Lu-Xia Ye, Yi-Qing Guo, Jun-Fei Liu, Hai-Hong Zheng, Dechao Feng, Wuran Wei

**Affiliations:** ^1^ Department of Urology, West China Hospital, Institute of Urology, Sichuan University, Chengdu, Sichuan, China; ^2^ Department of Pathology, Taizhou Hospital, Wenzhou Medical University, Linhai, Zhejiang, China; ^3^ Department of Urology, Sichuan Cancer Hospital and Institute, Chengdu, Sichuan, China

**Keywords:** endothelial cells, kidney renal clear cell carcinoma, single-cell, precision medicine, tumor microenvironment

## Abstract

**Background:** Endothelial cells in the tumor microenvironment play an important role in the development of kidney renal clear cell carcinoma (KIRC). We wanted to further identify the function of endothelial cells in KIRC patients by integrating single-cell and bulk RNA sequencing data.

**Methods:** Online databases provide the original data of this study. An endothelial-related prognostic index (ERPI) was constructed and validated by R version 3.6.3 and relative packages.

**Results:** The ERPI consisted of three genes (CCND1, MALL, and VWF). Patients with high ERPI scores were significantly correlated with worse prognosis than those with low ERPI scores in the TCGA training group, TCGA test group, and GSE29609 group. A positive correlation was identified between the ERPI score and poor clinical features. The results of functional analysis indicated that ERPI was significantly associated with immune-related activities. We suggested that patients with high ERPI scores were more likely to benefit from immunotherapy based on the results of immune checkpoints, tumor microenvironment, stemness index, and TCIA, while patients with low ERPI scores were sensitive to gemcitabine, docetaxel, paclitaxel, axitinib, pazopanib, sorafenib, and temsirolimus according to the results of the “pRRophetic” algorithm. Therefore, this ERPI may help doctors choose the optimal treatment for patients with KIRC.

**Conclusion:** By integrating single-cell and bulk RNA sequencing data from KIRC patients, we successfully identified the key genes from the perspective of endothelial cells in the tumor microenvironment and constructed ERPIs that had positive implications in precision medicine.

## Introduction

In 2020, there were 431,288 newly diagnosed kidney cancer patients worldwide, accounting for approximately 2.2% of all cancers (Siegel et al., 2021). In western countries, with the highest incidence occurring, kidney cancer accounts for 3% of all cancers (Capitanio et al., 2019). Of these, 80% of kidney cancer is kidney renal clear cell carcinoma (KIRC), which has a shorter survival time than kidney cancer with other kinds of pathologies (EAU Guidelines, 2022). Today, surgery is the main treatment for localized KIRC. However, many patients will relapse quickly, either after partial or radical nephrectomy (Escudier et al., 2019). Furthermore, approximately one-third of patients with localized RCC inevitably develop metastases (Escudier et al., 2019). To improve the prognosis of patients with KIRC, various treatments are clinically applied, such as chemotherapy, targeted therapy, radiotherapy, and immunotherapy. Nevertheless, the effectiveness of the current treatment is far from satisfactory. Under the guidance of precision medicine, doctors try to find new targets to cure KIRC and choose the optimal treatment for KIRC patients by screening powerful biomarkers (Yu et al., 2022).

KIRC is a tumor rich in angiogenesis (Edeline et al., 2012). At some time, angiogenesis plays a vital role in tumor progression (Folkman, 1972). Of these, vascular endothelial growth factor (VEGF) can promote the angiogenesis and cell proliferation of KIRC by activating endothelial cells, while tyrosine kinase inhibitors (TKIs) can inhibit the growth of KIRC cells by inhibiting VEGF signaling (Edeline et al., 2012; Martin et al., 2012). Nevertheless, many patients are resistant to TKIs (Marona et al., 2022). Meanwhile, the adverse effects of anti-VEGF agents are present in many patients (Chen and Cleck, 2009). Similarly, only some KIRC patients are sensitive to immunotherapy (Powles and ESMO Guidelines Committee, 2021). These therapeutic resistances may be explained by the tumor microenvironment (Kim et al., 2021; Feng et al., 2022; Gifre-Renom et al., 2022). Recently, single-cell RNA-sequencing (scRNA-seq) technologies have attracted wide attention and have allowed us to sequence and analyze thousands of cells per tumor (Schreibing and Kramann, 2022). Therefore, we can discuss the function of specific cell types in the tumor environment. For instance, by analyzing scRNA-seq, Zhang et al. (2021) reported that there was a negative correlation between endothelial cell infiltration and immunotherapy response.

Thus, by integrating single-cell and bulk RNA sequencing data, KIRC data from online databases were used to identify the key genes from the perspective of endothelial cells in the tumor microenvironment. Then, we constructed and validated an endothelial-related prognostic index. We also explored the predictive value of the index for immunotherapy, targeted therapy and chemotherapy.

## Methods

### Data collection

The expression and clinical data were extracted from the Cancer Genome Atlas (www.gdc.cancer.gov, TCGA) database. The “limma” package was used to identify the differentially expressed genes (|log2FoldChange| >1 and *p*-value < 0.05) between 539 KIRC samples and 72 normal samples. Then, we excluded KIRC patients with postoperative survival times shorter than 30 days or without survival outcomes. The included patients were randomly divided into the TCGA training group (*n* = 309) and TCGA test group (*n* = 206). As an external validation dataset, GSE29609 (Edeline et al., 2012) was downloaded from the Gene Expression Omnibus (https://www.ncbi.nlm.nih.gov, GEO) database.

The xCELL (https://xcell.ucsf.edu/) (Aran et al., 2017) website was employed to calculate the endothelial cell content of each included KIRC sample in the TCGA dataset. Then, based on the gene expression and endothelial cell content of each included TCGA sample, the endothelial-related genes (|coefficients| > 0.3 and *p*-value <0.05) were screened by Pearson correlation analysis. Then, the markers of endothelial cells (|log2FoldChange| >1 and *p*-value < 0.05) were extracted from Tumor Immune Single-cell Hub 2 (http://tisch.comp-genomics.org, TISCH2) (Sun et al., 2021), which is a single-cell RNA-seq database and provides markers of cell type by validating differential genes between different cell types in the tumor microenvironment. In our study, GSE139555 provided the original data to analyze in TISCH2 (Wu et al., 2020).

### Construction of the endothelial-related prognostic index

The “VennDiagram” package was utilized to screen the differentially coexpressed endothelial-related genes. Then, by following a minimum standard of 10-fold cross-validation, the lasso regression model applied penalties to these genes based on the TCGA training group. The prognostic value of the selected genes in the lasso regression model was assessed by a univariate Cox regression model. Finally, three genes (CCND1, MALL and VWF) were used to construct the endothelial-related prognostic index score (ERPIs). Meanwhile, GeneMANIA (www.genemania.org) (Warde-Farley et al., 2010) was used to evaluate the interacting proteins of these three genes.

### Validation of the clinical value of ERPI

Before estimating the ERPI, we assessed the prognostic value of endothelial cells in KIRC patients using Kaplan‒Meier curves. Then, the prognostic value of the ERPI was validated by Kaplan‒Meier curves in the TCGA training group, TCGA test group, and GSE29609 dataset. Furthermore, we also evaluated the prognostic value of ERPI in clinical subgroups using Kaplan‒Meier curves. The correlation between ERPI and clinical parameters was assessed by the Wilcoxon rank-sum test. Moreover, according to the results of the univariable Cox regression model, factors with a *p*-value < 0.05 were included in the multivariable Cox regression model to estimate the independent prognostic value of ERPI. Referring to the results of the multivariable Cox regression model, we established two nomograms based on the TCGA training group and TCGA test group. The performance of these two nomograms was evaluated by the concordance index (C-index), multiparameter ROC analysis, and calibration curves.

### Functional analysis

We performed Gene Ontology (GO) enrichment analysis and Kyoto Encyclopedia of Genes and Genomes (KEGG) enrichment analysis based on the TCGA dataset (including TCGA training group and TCGA test group) and ERPI. The GO results and enriched KEGG pathways were screened out with *p*-value <0.05 and Q < 0.05 criteria. Meanwhile, Gene Set Enrichment Analysis (GSEA) was used to screen REACTOME pathways with *p*-value <0.05 and FDR<25%.

The role of ERPI in immune-related analysis was also explored because the functional results indicated that ERPI was involved in immune-related activities. The immune checkpoints were compared between the high- and low-ERPI groups. In the tumor microenvironment, according to the results of xCELL, the Wilcoxon rank-sum test was utilized to compare the contents of infiltrated immune cells in the high- and low-ERPI groups. Moreover, the one-class logistic regression machine learning algorithm contributed an mRNA expression‐based stemness index (mRNAsi), which could reflect the cell stemness of samples (Malta et al., 2018). Thus, we calculated the mRNAsi score of each sample using this algorithm and compared the mRNAsi score between the high- and low-ERPI groups. To predict the response to immunotherapy, the Cancer Immunome Atlas (https://www.tcia.at/home, TCIA) (Charoentong et al., 2017), a database providing comprehensive immunogenomic analyses based on the TCGA database, was used to evaluate the immunotherapy response in each sample. After that, the TCIA score was compared between the high- and low-ERPI groups. Furthermore, since tumor-related endothelial cells can induce chemotherapy resistance and angiogenesis, the predictive value of ERPI was evaluated by the “pRRophetic” package. The half-maximal inhibitory concentration (IC50) was the main endpoint of chemotherapy and targeted therapy, which was also compared between the high- and low-ERPI groups.

### Statistical analysis

According to the normality and quality of variances of the data, one-way ANOVA or the Mann‒Whitney *U* test was used to perform statistical analysis of three or more continuous variables. Quantitative data in two groups were compared using Student’s t-test. All analyzed data are displayed as the standard deviation (SD). A *p* < 0.05 was considered significant for all analyses, which were performed using R version 3.6.3 and relative packages.

## Results

### Construction of the index and basic data

**FIGURE 1:**
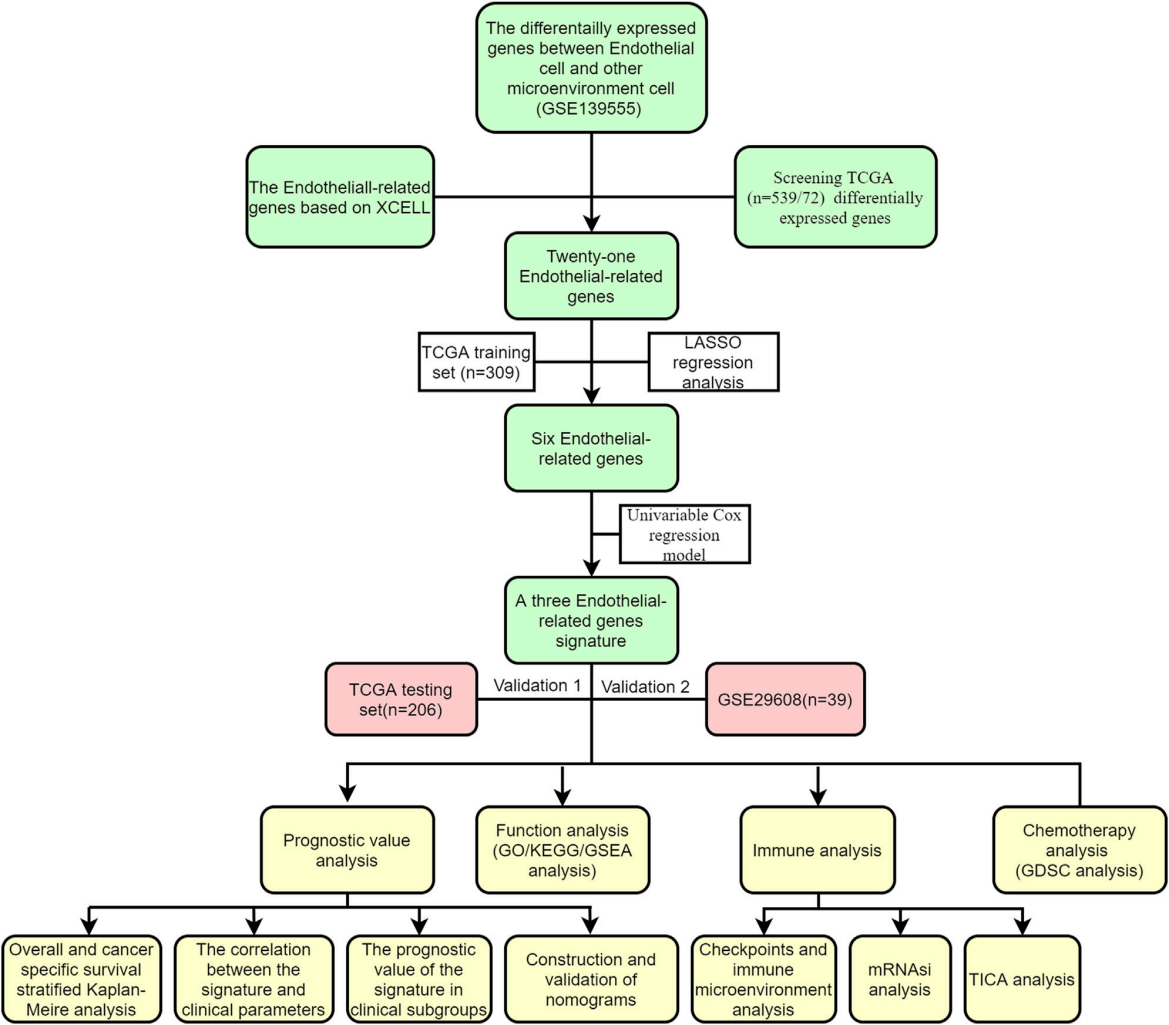
The workflow of this study.

**FIGURE 2:**
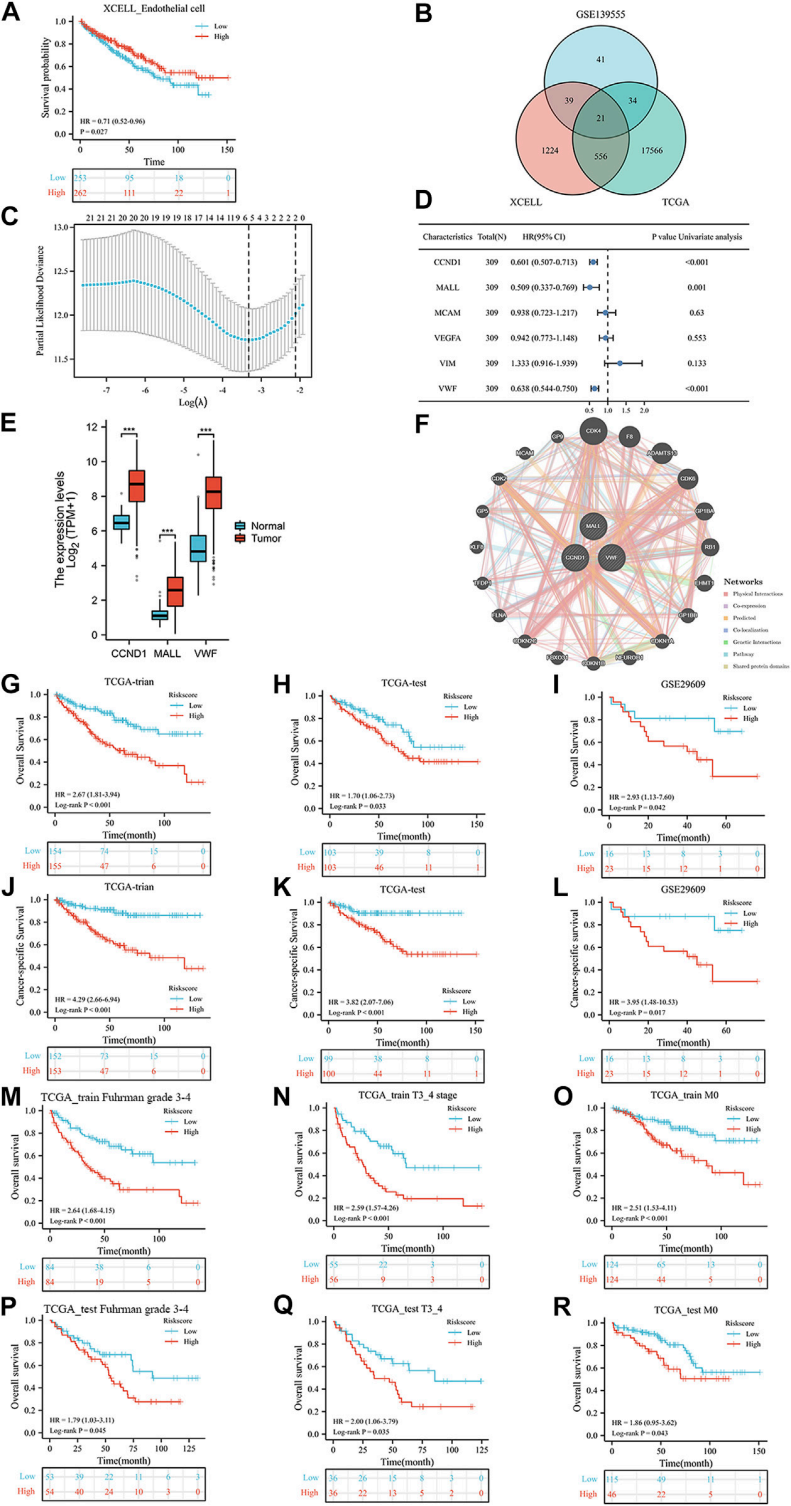
The prognostic value of endothelial cells in TCGA dataset **(A)**, the co-expressed genes **(B)**, the cross-validation to determine the optimal penalty parameter lambda **(C)**, the prognostic value of these six genes in overall survival (OS) according to the results of univariable Cox regression analysis in TCGA training group **(D)**, the expression of these three genes **(E)**, the protein–protein interaction network **(F)**, the Kaplan-Meier analysis results of OS in TCGA training group **(G)**, TCGA test group **(H)**, and GSE29609 dataset **(I)**; the Kaplan-Meier analysis results of cancer-specific survival in TCGA training group **(J)**, TCGA test group **(K)**, and GSE29609 dataset **(L)**; the Kaplan-Meier analysis results of OS in TCGA training subgroups: Furhman grade3-4 **(M)**, T3_4 stage **(N)**, and no distant metastasis **(O)** subgroups; the Kaplan-Meier analysis results of OS in TCGA test subgroups: Furhman grade3-4 **(P)**, T3_4 stage **(Q)**, and no distant metastasis **(R)** subgroups.

Figure 1 shows the workflow of our work. After calculating the endothelial cell content in each TCGA sample, Kaplan‒Meier curves indicated that patients with low endothelial cell infiltration had worse overall survival (OS) than those with high endothelial cell infiltration (Figure 2A, *p* = 0.027). A total of 1840 endothelial-related genes were confirmed by Pearson correlation analysis (Supplementary Table S1). There were 135 endothelial-specific genes acquired from TISCH2 (Supplementary Table S2). The ‘VennDiagram’ package identified 21 differentially coexpressed endothelial-related genes (Figure 2B), and the lasso regression analysis ultimately selected six genes (Figure 2C). CCND1, MALL and VWF exhibited significant prognostic value in the TCGA training group (Figure 2D). Meanwhile, CCND1, MALL and VWF were highly expressed in KIRC (Figure 2E). The CDK4 protein was the interacting protein of these three genes (Figure 2F). As described in the methods section, the ERPI consisted of CCND1, MALL and VWF. After calculating the ERPI score, we divided patients in the TCGA training group and TCGA test group into low- and high-ERPI groups according to the median value of ERPI. Due to the limited patient number of the GSE29609 dataset, we employed ROC curves to find the optimal cutoff value of the ERPI score, which was 3.9373. Patients with ERPI scores higher than 3.9373 were divided into the high-ERPI group, while the remaining patients were divided into the low-ERPI group. In the TCGA training group, patients in the high-ERPI group were positively correlated with poor clinical features, such as Fuhrman grade, American Joint Committee on Cancer (AJCC) stage, and T stage (Table 1). Detailed information on the TCGA test group and GSE29609 is presented in Supplementary Tables S3, S4, respectively.

**TABLE 1:** Clinicopathologic characteristics of the TCGA training dataset.

Characteristic	Low risk-score	High risk-score	*p*
n	154	155	
Age, mean ± SD	60.68 ± 11.87	61.28 ± 11.89	0.653
Gender, n (%)			0.377
Female	56 (18.1%)	48 (15.5%)	
Male	98 (31.7%)	107 (34.6%)	
Fuhrman grade, n (%)			<0.001
Grade 1_2	88 (28.9%)	49 (16.1%)	
Grade 3_4	65 (21.3%)	103 (33.8%)	
AJCC stage, n (%)			<0.001
AJCC Stage I_II	112 (36.5%)	76 (24.8%)	
AJCC stage III_IV	41 (13.4%)	78 (25.4%)	
Distant metastasis, n (%)			0.006
No	132 (45.2%)	116 (39.7%)	
Yes	13 (4.5%)	31 (10.6%)	
Lymph node metastasis, n (%)			0.034
N0	72 (48%)	69 (46%)	
Yes	1 (0.7%)	8 (5.3%)	
T stage, n (%)			<0.001
T1_2	114 (36.9%)	83 (26.9%)	
T3_4	40 (12.9%)	72 (23.3%)	
Overall survival, n (%)			<0.001
Alive	123 (39.8%)	84 (27.2%)	
Dead	31 (10%)	71 (23%)	
Cancer-specific survival, n (%)			<0.001
Alive	139 (45.6%)	99 (32.5%)	
Dead	14 (4.6%)	53 (17.4%)	

AJCC, american joint committee on cancer; SD, standard deviation; n, Number.

### ERPI has clinical value

In the results of Kaplan‒Meier curves, patients with high ERPI scores were significantly associated with worse OS than those with low ERPI scores in the TCGA training group (Figure 2G, *p* < 0.001), TCGA test group (Figure 2H, *p* = 0.033), and GSE29609 (Figure 2I, *p* = 0.042) dataset. Regarding cancer-specific survival, compared with patients with low ERPI scores, shorter survival times were observed in patients with high ERPI scores in the TCGA training group (Figure 2J, *p* < 0.001), TCGA test group (Figure 2K, *p* < 0.001), and GSE29609 (Figure 2L, *p* = 0.017) dataset. In TCGA training subgroups, patients in the high-ERPI group had worse OS than those in the low-ERPI group, including Fuhrman grade 3–4 (Figure 2M, *p* < 0.001), T stage 3_4 (Figure 2N, *p* < 0.001), and no distant metastasis (Figure 2O, *p* < 0.001) subgroups. In TCGA training subgroups, shorter OS time was significantly associated with patients in the high-ERPI group than those in the low-ERPI group, such as Fuhrman grade 3–4 (Figure 2P, *p* = 0.045), T stage 3_4 (Figure 2Q, *p* = 0.035), and no distant metastasis (Figure 2R, *p* = 0.043) subgroups. The prognostic value of ERPI in GSE29609 subgroups was not identified due to the limited number of patients.

**FIGURE 3:**
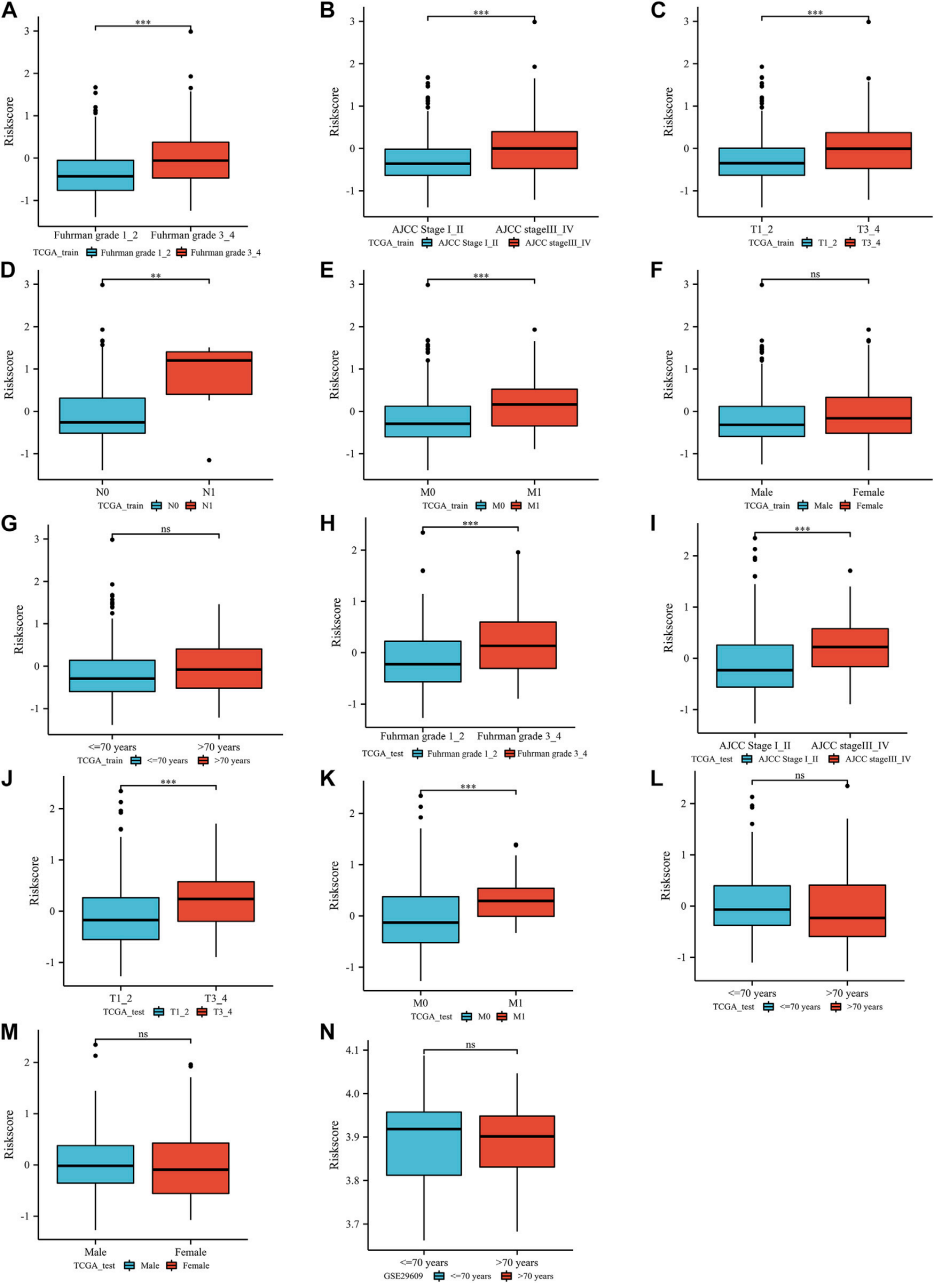
The correlation between the index and clinical parameters: TCGA training group (Furhman grade **(A)**, AJCC stage **(B)**, T stage **(C)**, lymph node metastasis stage **(D)**, distant metastasis stage **(E)**, sex **(F)**, age **(G)**), TCGA test group (Furhman grade **(H)**, AJCC stage **(I)**, T stage **(J)**, distant metastasis stage **(K)**, age **(L)**, sex **(M)**), GSE29609 dataset (age **(N)**). N: lymph node metastasis; M: distant metastasis; AJCC: American Joint Committee on Cancer; ns, *p* ≥ 0.05; *, *p* < 0.05; **, *p* < 0.01; ***, *p* < 0.001.

In the TCGA training group, a positive correlation was identified between the ERPI score and poor clinical features, such as Fuhrman grade (Figure 3A), AJCC stage (Figure 3B), T stage (Figure 3C), lymph node metastasis (Figure 3D), and distant metastasis (Figure 3E). Fortunately, there was no difference in ERPI between patients of different sexes (Figure 3F) and ages (Figure 3G). Similarly, in the TCGA-test group, ERPI was also positively correlated with poor clinical characteristics, including Fuhrman grade (Figure 3H), AJCC stage (Figure 3I), T stage (Figure 3J), and distant metastasis (Figure 3K). There was also no difference in ERPI between patients of different ages (Figure 3L) and sexes (Figure 3M) in the TCGA-test group. In the GSE29609 dataset, there was no difference between ERPI and age (Figure 3N). These results indicated that the ERPI score was positively associated with poor clinical features and that there was no difference between patients of different ages and sexes.

### Construction and validation nomogram

**FIGURE 4:**
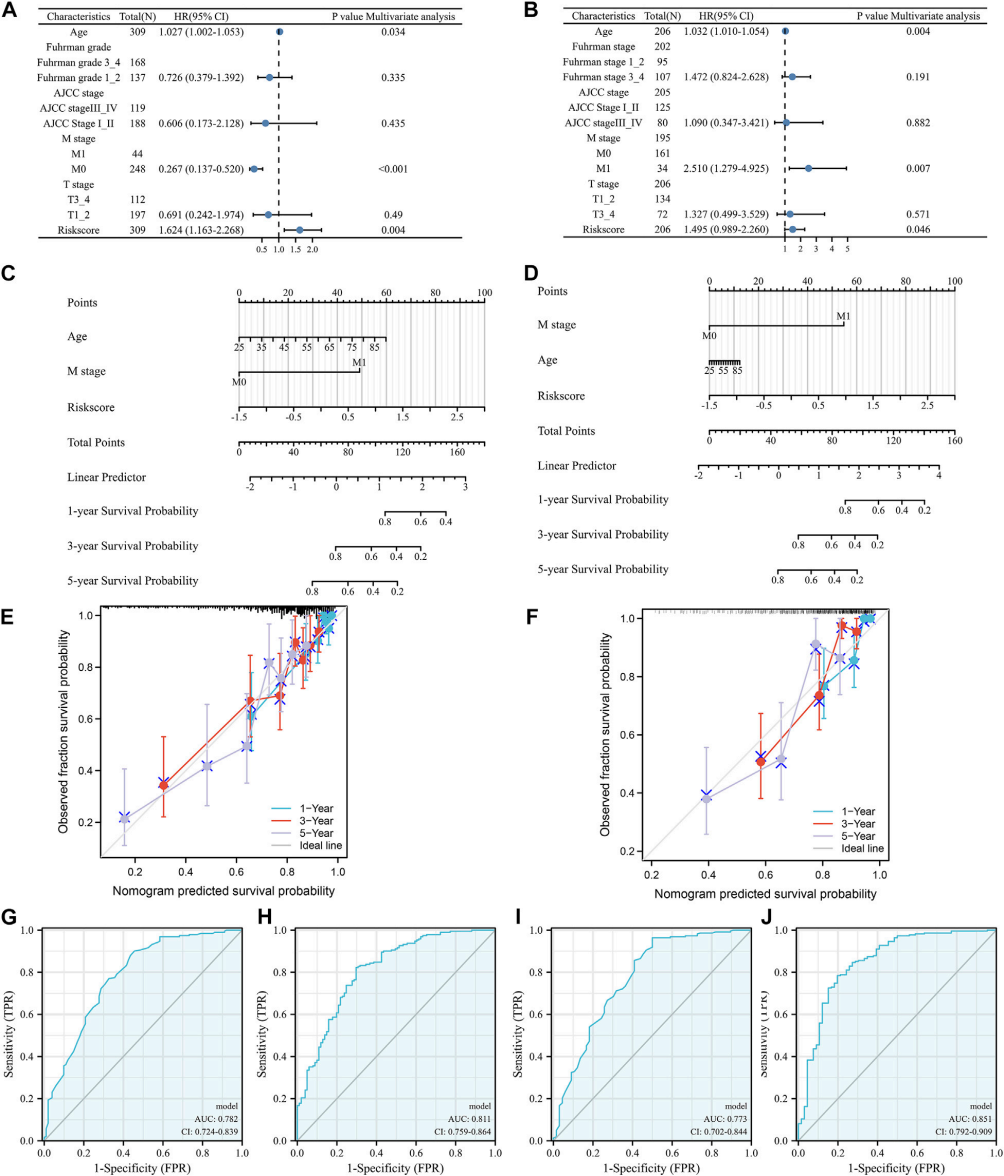
Validation of the independent prognostic value of the index: multivariable Cox regression model in TCGA training group **(A)** and TCGA test group **(B)**, nomograms of TCGA training group **(C)** and TCGA test group **(D)**, calibration plots of TCGA training nomogram **(E)** and TCGA test nomogram **(F)**, multiparameter ROC analysis without **(G)**/with **(H)** the index in TCGA training nomogram, multiparameter ROC analysis without **(I)**/with **(J)** the index in TCGA test nomogram, N: lymph node metastasis; M: distant metastasis; AJCC: American Joint Committee on Cancer.

According to the results of the univariable Cox regression model, we constructed a multivariable Cox regression model that consisted of age, Furhman grade, AJCC stage, M stage, T stage, and ERPI score based on the TCGA training group. In the results, ERPI presented independent prognostic value (Figure 4A, *p* = 0.004). In the TCGA test group, ERPI could also independently predict the prognosis of BC patients (Figure 4B, *p* = 0.046). Referring to the results of multivariable analysis, we established two nomograms based on the TCGA training group (Figure 4C) and TCGA test group (Figure 4D). The C-index of the TCGA training group nomogram was 0.756 (0.730–0.782). The nomogram of the TCGA test group also had a moderate C-index, which was 0.744 (0.713–0.775). The predicted values in the two nomograms (TCGA training group (Figure 4E) and TCGA test group (Figure 4F)) fluctuated around the true values. Furthermore, in the nomogram of the TCGA training group, the area under the curve (AUC) value increased from 0.782 to 0.811 after adding the ERPI into the model (Figures 4G,H). This phenomenon was also observed in the nomogram of the TCGA test group. After adding ERPI to the model, the AUC value increased from 0.773 to 0.851 (Figures 4I,J). These results indicated that the ERPI had significant prognostic value for BC patients.

### ERPIs is ERPIinvolved in immune-related activities

**FIGURE 5:**
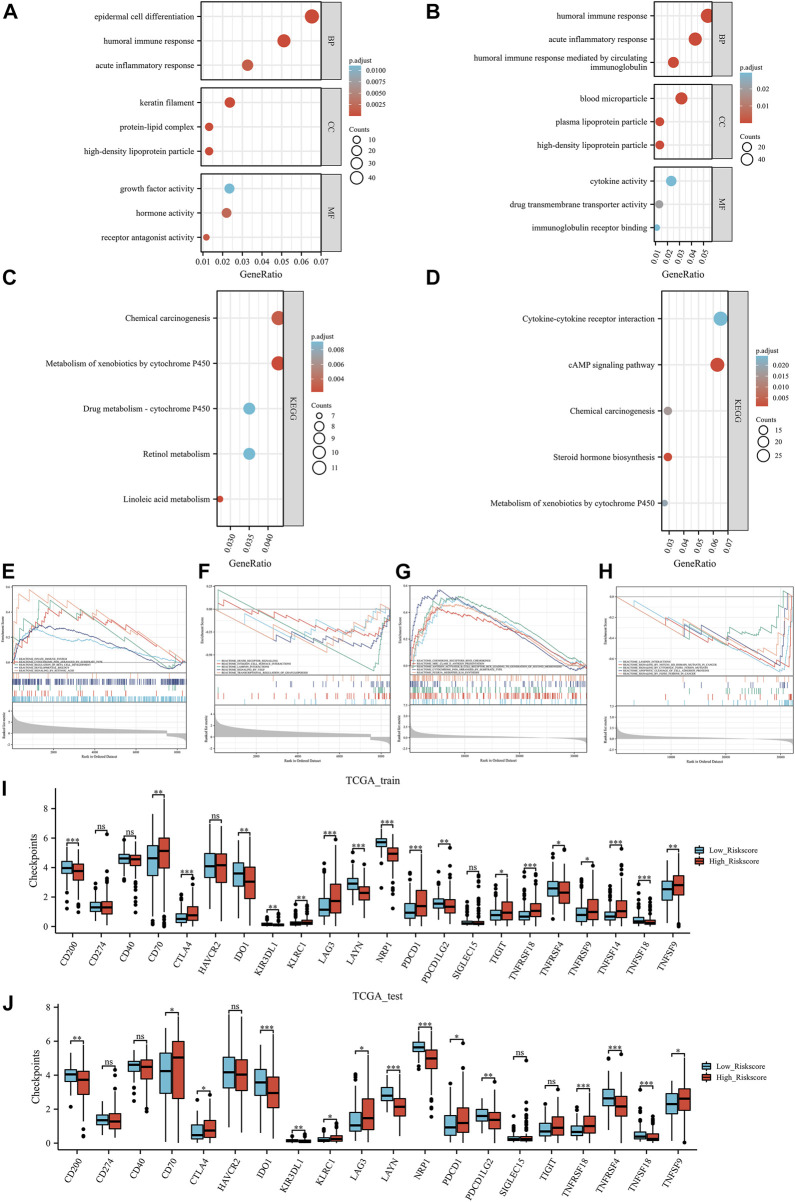
Functional analysis: the Gene Ontology results of TCGA training group **(A)** and TCGA test group **(B)**, Kyoto Encyclopedia of Genes and Genomes results of TCGA training group **(C)** and TCGA test group **(D)**, Gene Set Enrichment Analysis results TCGA training group **(E, F)** and TCGA test group **(G, H)**. Immune checkpoint results of the TCGA training group **(I)** and TCGA test group **(J)**. Ns, *p* ≥ 0.05; *, *p* < 0.05; **, *p* < 0.01; ***, *p* < 0.001.

As shown in Figure 5A, based on the data of TCGA training group, GO enriched epidermal cell differentiation biological process, humoral immune response biological process, growth factor activity, and hormone activity. In the TCGA test group, ERPI was involved in acute inflammatory response biological processes, humoral immune response biological processes, drug transmembrane transporter activity, and cytokine activity (Figure 5B). In terms of pathways, based on the data of TCGA training group, KEGG enriched chemical carcinogenesis pathway and metabolism-related pathways (Figure 5C). In the TCGA test group, ERPI was involved in chemical carcinogenesis, metabolism of xenobiotics by cytochrome P450, and cytokine‒cytokine receptor interaction pathways (Figure 5D).

In further GSEA, based on the data of the TCGA training group, GSEA enriched the innate immune system, cytochrome p450 arranged by substrate type, integrin cell surface interactions, and signaling by VEGF pathways (Figures 5E,F). In the TCGA test group, ERPI involved in chemokine receptors binds chemokines, MHC class II antigen presentation, apoptotic cleavage of cell adhesion proteins, and signaling by FGFR3 fusions in cancer pathways (Figures 5G,H). Taken together, these results suggested that ERPI was associated with endothelial cells and involved in immune-related activities and pathways.

### ERPI could predict immunotherapy and chemotherapy responses

**FIGURE 6:**
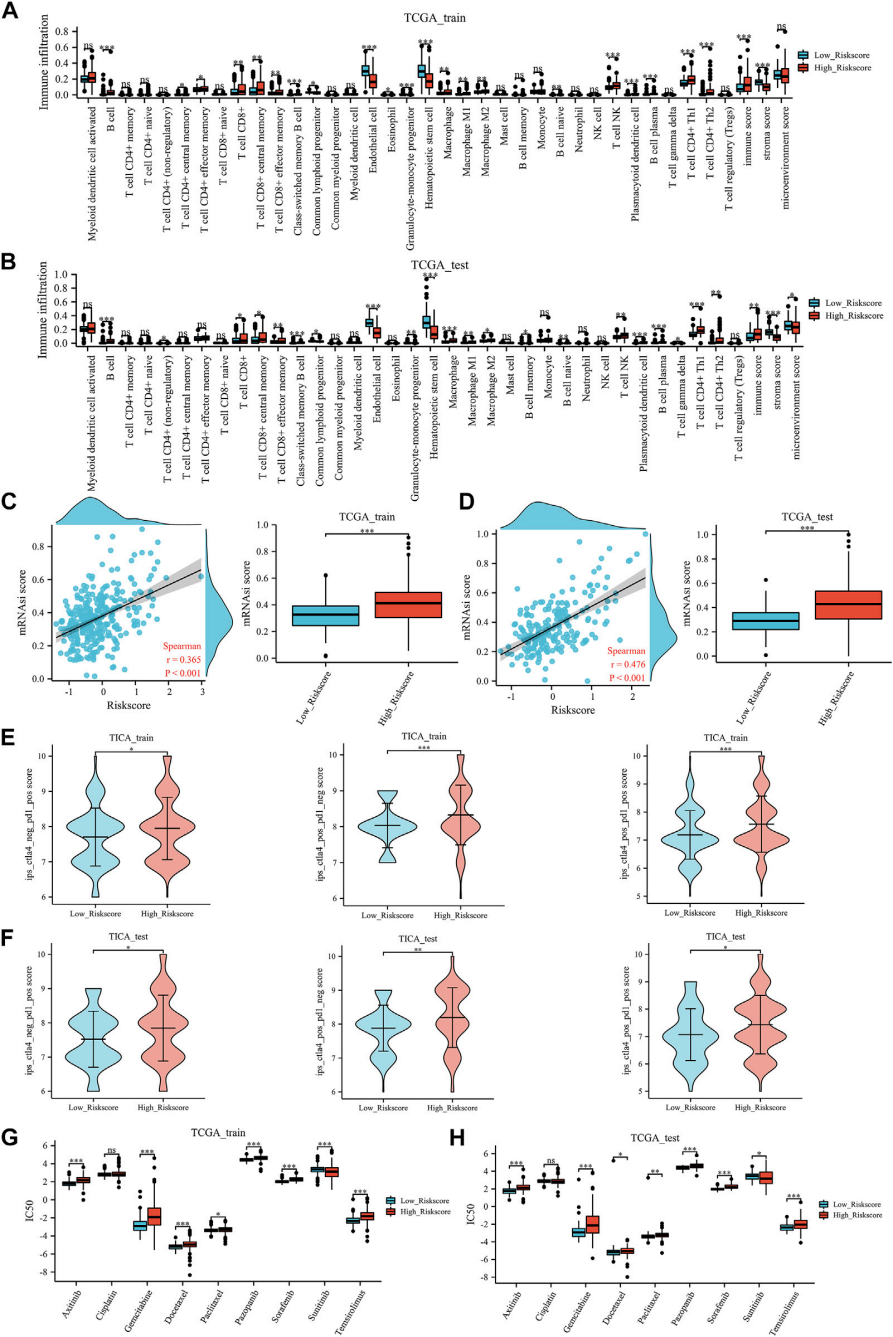
Immune-related analysis: the tumor microenvironment results of TCGA training group **(A)** and TCGA test group **(B)**, the stemness index results of TCGA training group **(C)** and TCGA test group **(D)**, the TCIA results of TCGA training group **(E)** and TCGA test group **(F)**, the prediction of chemotherapy and targeted therapy in TCGA training group **(G)** and TCGA test group **(H)**. mRNAsi: stemness index; IC50: the half-maximal inhibitory concentration. Ns, *p* ≥ 0.05; *, *p* < 0.05; **, *p* < 0.01; ***, *p* < 0.001.

In both the TCGA training and TCGA test groups, compared with the low ERPI group, the high ERPI group had higher immune checkpoint expression, such as CTLA4, PDCD1, and KLRC1 (Figures 5I,J). In the tumor environment, Figures 6A,B show that samples in the high ERPI group had higher B cell, CD8^+^ T cell, macrophage cell, NK T cell, and CD4^+^ Th1/2 cell infiltration, while samples in the low ERPI group had higher endothelial cell infiltration in both the TCGA training and TCGA test groups.

Furthermore, in the TCGA training group (Figure 6C) and the TCGA test group (Figure 6D), a positive correlation was identified between the mRNAsi score and ERPI score. Meanwhile, patients with high ERPI scores had significantly higher mRNAsi scores than those with low ERPI scores.

In the field of immunotherapy prediction, TICA results indicated that patients with high ERPI scores were more likely to benefit from immunotherapy (Figure 6E) in the TCGA training group. Similarly, in the TCGA test group, patients in the high ERPI group had a higher response to immunotherapy than those in the low ERPI group (Figure 6F). In chemotherapy and targeted therapy, patients with low ERPI scores had significantly lower IC50 values of gemcitabine, docetaxel, paclitaxel, axitinib, pazopanib, sorafenib, and temsirolimus, which might suggest that patients in the low-ERPI group were more likely to benefit from chemotherapy and targeted therapy in both the TCGA training group (Figure 6G) and TCGA test group (Figure 6H).

## Discussion

Endothelial cells in the tumor environment play a key role in antiangiogenic therapy, which is a main therapy for KIRC (Gifre-Renom et al., 2022). Meanwhile, Zhang et al. (Zhang et al., 2021) reported that there was a negative correlation between endothelial cell infiltration and immunotherapy response. These results identified the important role of endothelial cells in KIRC. Therefore, in this study, for the first time, we constructed an endothelial-related prognostic index and validated the clinical value of the index. Furthermore, the index could also predict the response to immunotherapy, chemotherapy, and targeted therapy, thereby providing a useful tool for clinical use.

MAL-like protein (MALL) is normally expressed in endothelial cells and can encode an element of the machinery for raft-mediated trafficking in endothelial cells. In the early stage of carcinogenesis, the expression of MALL changes (Jiang et al., 2009). Cyclin D1 (CCND1), as a protein encoding gene, is highly expressed in KIRC and can promote KIRC cell proliferation by regulating the cell cycle (Musgrove et al., 2011; Patel et al., 2022). With VEGF stimulation, endothelial cells can release Von Willebrand Factor (VWF), which encodes a glycoprotein involved in hemostasis (Maisonpierre et al., 1997). There was a positive correlation between the tumor burden of metastatic renal cell cancer and VWF expression (van der Veldt et al., 2012). Among the interacting proteins, cyclin-dependent kinase 4 (CDK4) is a member of the Ser/Thr protein kinase family and forms a complex with CCND1 to regulate the cell cycle G1/S transition (Adamopoulos et al., 2022). Together, these results indicated that these three genes were associated with endothelial cells and involved in the development of tumors, especially KIRC.

According to the Kaplan‒Meier curves, KIRC patients with high endothelial cell infiltration had a better prognosis than those with low endothelial cell infiltration. Patients in the low-ERPI group had higher endothelial cell infiltration. Meanwhile, patients in the low-ERPI group were significantly associated with better prognosis in the TCGA training group, TCGA test group, and GSE32894 dataset, which was consistent with the finding that high endothelial cell infiltration in KIRC was significantly correlated with good prognosis. This finding was also reported by Zhang et al. (2021) who also found that KIRC patients with high endothelial cell content tend to have better OS than those with low endothelial cell content. Interestingly, they found that patients with high endothelial cell content had significantly better OS than those with high CD8^+^ T-cell content. This also agrees with our earlier observations, which showed that patients in the low ERPI group had low CD8^+^ T-cell and high endothelial cell infiltration and were associated with good OS. Based on these results, we established and validated two nomograms with clinical value. Furthermore, ERPI was statistically correlated with poor clinical features. These results indicated that the ERPI had clinical value for KIRC patients.

In the results of functional analysis, ERPI was associated with cellular components (such as keratin filaments and blood microparticles) and enriched signaling by Notch1 HD domain mutants in cancer and signaling by VEGF pathways. Of these, keratin filaments are required for maintaining the mechanical stability of epithelial cells (Nafeey et al., 2016). In pathways, Clark et al. (Clark et al., 2019) observed that the highest endothelial cell signature group also enriched the Notch signaling pathway. These functional results repeatedly identified that ERPI was truly associated with endothelial cells in the tumor microenvironment. In addition, functional analysis indicated that ERPI is involved in immune-related pathways. Thus, we explored the role of ERPI in immune-related analysis. Regarding immune checkpoints, the high ERPI group had higher CTLA4, PDCD1, and KLRC1 expression than the low ERPI group. However, we could not suggest which group might have a higher response rate to immunotherapy because these checkpoints could not predict the response to immunotherapy (Labadie et al., 2021). It remained us that the immune environment was different between the high- and low-ERPI groups. In terms of the tumor microenvironment, tissues in the high ERPI group had higher CD8^+^ T-cell infiltration and less endothelial cell infiltration. In KIRC, CD8^+^ T cells activated by anti-CTLA4 immunotherapy could kill tumor cells (Yang et al., 2007). In another study, tumor-infiltrating CD8^+^ T cells were positively associated with a response to anti-PD1 therapy (Borcherding et al., 2021). Meanwhile, in metastatic KIRC, low endothelial cell infiltration was correlated with a better response to immunotherapy (Zhang et al., 2021). Given the results of the above studies, we reasonably speculated that patients in the high-ERPI group were more likely to benefit from immunotherapy. In stemness index analysis, patients with high mRNAsi scores had a higher response to immunotherapy than those with low mRNAsi scores (Malta et al., 2018). Consistent with the results of the tumor microenvironment, a higher mRNAsi score was identified in patients with a high ERPI score, which suggested that patients in the high-ERPI group were more likely to benefit from immunotherapy. In further TICA analysis, patients in the high-ERPI group had a higher response to anti-CTLA4 and anti-PD1 therapies than patients in the low-ERPI group. As an anticancer therapy, antiangiogenic therapy improves the prognosis of patients with KIRC (Ferrara et al., 2005). Nevertheless, only some patients could benefit from the therapy (Marona et al., 2022). To help solve this problem, we also explored the predictive value of ERPI in targeted therapy and chemotherapy. Compared with the high-ERPI group, patients in the low-ERPI group were sensitive to many targeted therapies and chemotherapies. Taken together, these results indicated that ERPI could predict the response to immunotherapy, targeted therapy and chemotherapy.

## Conclusion

By integrating single-cell and bulk RNA sequencing data from KIRC patients, we successfully identified the key genes from the perspective of endothelial cells in the tumor microenvironment and constructed ERPIs that had positive implications in precision medicine.

## Data Availability

The original contributions presented in the study are included in the article/Supplementary Material, further inquiries can be directed to the corresponding authors.

## Abbreviations

AJCC, American Joint Committee on Cancer; CCND1, Cyclin D1; CDK4, Cyclin-dependent kinase 4; CC, Cellular Component; ERPI, Endothelial-related prognostic index; GEO, Gene Expression Omnibus; GO, Gene Ontology; IC50, The half-maximal inhibitory concentration; KEGG, Kyoto Encyclopedia of Genes and Genomes; KIRC, Kidney renal clear cell carcinoma; MALL, MAL-like protein; OS, Overall survival; SD, Standard deviation; TKIs, Tyrosine kinase inhibitors; TCIA, The Cancer Immunome Atlas; TISCH2, Tumor Immune Single-cell Hub 2; TCGA, The Cancer Genome Atlas; VEGF, Vascular endothelial growth factor; VWF, Von willebrand factor; BP, Biological Process; MF, Molecular Function.
