# Supplementary material for: A novel endothelial-related prognostic index by integrating single-cell and bulk RNA sequencing data for patients with kidney renal clear cell carcinoma
Source: Front Genet. 2023 Mar 10;14:1096491. doi: 10.3389/fgene.2023.1096491 (PMC10036355; doi:10.3389/fgene.2023.1096491)
Supplement: Supplementary file 1 [file DataSheet4.docx]

Supplementary table 4. The Clinicopathologic characteristics of the GSE29609 dataset.

| Characteristic | Low Risk-score | High Risk-score | p |
| --- | --- | --- | --- |
| n | 23 | 16 |  |
| Age, mean ± SD | 59.04 ± 14.66 | 64.75 ± 8.83 | 0.139 |
| T stage, n (%) |  |  | 1.000 |
| T1_2 | 9 (23.1%) | 7 (17.9%) |  |
| T3-4 | 14 (35.9%) | 9 (23.1%) |  |
| Lymph node metastasis, n (%) |  |  | 0.432 |
| N+ | 6 (15.4%) | 2 (5.1%) |  |
| N0 | 17 (43.6%) | 14 (35.9%) |  |
| Distant metastasis, n (%) |  |  | 0.317 |
| M0 | 13 (33.3%) | 12 (30.8%) |  |
| M1 | 10 (25.6%) | 4 (10.3%) |  |
| Fuhrman grade, n (%) |  |  | 0.752 |
| Grade1_2 | 13 (33.3%) | 8 (20.5%) |  |
| Grade3_4 | 10 (25.6%) | 8 (20.5%) |  |
| Overall survival, n (%) |  |  | 0.099 |
| Alive | 10 (25.6%) | 12 (30.8%) |  |
| Dead | 13 (33.3%) | 4 (10.3%) |  |
| Cancer-specific survival, n (%) |  |  | 0.024 |
| Alive | 10 (25.6%) | 13 (33.3%) |  |
| Dead | 13 (33.3%) | 3 (7.7%) |  |

SD: Standard deviation; n: Number.
