# Supplementary material for: A novel endothelial-related prognostic index by integrating single-cell and bulk RNA sequencing data for patients with kidney renal clear cell carcinoma
Source: Front Genet. 2023 Mar 10;14:1096491. doi: 10.3389/fgene.2023.1096491 (PMC10036355; doi:10.3389/fgene.2023.1096491)
Supplement: Supplementary file 2 [file DataSheet3.docx]

Supplementary table 3. The clinicopathologic characteristics of the TCGA test set.

| Characteristic | Low Risk-score | High Risk-score | p |
| --- | --- | --- | --- |
| n | 103 | 103 |  |
| Age, mean ± SD | 58.7 ± 13.33 | 60.26 ± 11.89 | 0.375 |
| Gender, n (%) |  |  | 0.560 |
| Female | 39 (18.9%) | 34 (16.5%) |  |
| Male | 64 (31.1%) | 69 (33.5%) |  |
| Fuhrman grade, n (%) |  |  | 0.016 |
| Grade 1_2 | 57 (28.2%) | 38 (18.8%) |  |
| Grade 3_4 | 45 (22.3%) | 62 (30.7%) |  |
| AJCC stage, n (%) |  |  | < 0.001 |
| AJCC Stage I_II | 75 (36.6%) | 50 (24.4%) |  |
| AJCC stage III_IV | 28 (13.7%) | 52 (25.4%) |  |
| Distant metastasis, n (%) |  |  | 0.001 |
| No | 89 (45.6%) | 72 (36.9%) |  |
| Yes | 8 (4.1%) | 26 (13.3%) |  |
| Lymph node metastasis, n (%) |  |  | 0.454 |
| No | 41 (42.7%) | 48 (50%) |  |
| Yes | 2 (2.1%) | 5 (5.2%) |  |
| T stage, n (%) |  |  | 0.005 |
| T1_2 | 77 (37.4%) | 57 (27.7%) |  |
| T3_4 | 26 (12.6%) | 46 (22.3%) |  |
| Overall survival, n (%) |  |  | 0.003 |
| Alive | 79 (38.3%) | 58 (28.2%) |  |
| Dead | 24 (11.7%) | 45 (21.8%) |  |
| Cancer-specific survival, n (%) |  |  | < 0.001 |
| Alive | 91 (45.7%) | 67 (33.7%) |  |
| Dead | 8 (4%) | 33 (16.6%) |  |

AJCC: American Joint Committee on cancer; SD: Standard deviation; n: Number.
